# Pre-exposure prophylaxis persistence at two sites in an integrated primary health care programme in South Africa

**DOI:** 10.3389/fpubh.2025.1460180

**Published:** 2025-05-12

**Authors:** Nosipho Shangase, Anele Jiyane, Fezile Buthelezi, Cara O’Connor, Ben Brown, Kate Rees

**Affiliations:** ^1^Anova Health Institute, Johannesburg, South Africa; ^2^Department of Community Health, School of Public Health, University of the Witwatersrand, Johannesburg, South Africa

**Keywords:** prevention, PrEP, retention, continuation, sub-Saharan Africa, implementation

## Abstract

**Introduction:**

HIV continues to be a public health concern and pre-exposure prophylaxis (PrEP) has become an important HIV prevention strategy. We examined PrEP persistence over time in two health facilities in Gauteng, South Africa.

**Methods:**

We conducted a retrospective analysis of PrEP persistence between 2018 and 2022 in two government health facilities in Gauteng. Data was manually extracted from the patient medical records and captured into REDCap. We defined PrEP persistence as PrEP use over time from the date of PrEP initiation without an interruption of more than 30 days between prescription refills. We used Kaplan–Meier survival curves to illustrate time to PrEP discontinuation. We used Cox proportional hazard models to examine factors associated with PrEP discontinuation.

**Results:**

In total, 344 patients were included in the analysis of which 48.2% were >24 years and 68.0% were females. PrEP persistence was 65.7% at month 1, 45.9% at month 2 and 37.8% at month 3 with a median persistence time of 76 days. Individuals >24 years were less likely to discontinue PrEP (aHR = 0.55, 95%CI: 0.39–0.77) compared to those 15–19-years. PrEP discontinuation was more likely in Facility A than in Facility B (aHR = 2.96, 95%CI: 2.10–4.17).

**Conclusion:**

We have shown that most people stop taking PrEP before their second PrEP refill and individuals >24 years had longer PrEP persistence than individuals aged 15–19 years. Service delivery factors appear to have a substantial effect on PrEP persistence. Health facilities should continue to make PrEP accessible by integrating PrEP with existing services to promote PrEP persistence.

## 1 Introduction

Worldwide, HIV continues to be a public health concern. Globally, there were 1.3 million new HIV infections in 2022 (1), 160,000 of which were in South Africa (2). In sub-Saharan Africa, 63% of new HIV infections were among women and girls (1). The expansion of the antiretroviral therapy (ART) programme has played an important role in preventing HIV transmission, with adherence to ART leading to an undetectable viral load and making HIV untransmittable (3). However, additional prevention measures are required. Pre-exposure prophylaxis (PrEP) is an important HIV prevention strategy (4), but uptake has not met expectations.

The World Health Organization (WHO) initially recommended that PrEP be offered to men who have sex with men (MSM) in 2014, followed by the 2015 expansion of PrEP recommendations to include all people at substantial risk of HIV (5). In response, South Africa introduced PrEP to a small number of sites in 2016 (6). The WHO further broadened PrEP recommendations in 2016 to include PrEP for pregnant and breastfeeding women (7). Since then, according to the South African National Department of Health (NDoH) PrEP 2020 guidelines, PrEP should be available at all primary health care facilities to all persons at substantial risk of HIV infection, including those requesting PrEP (8). Individuals are considered to be at substantial risk if they have: (1) sex without a condom, (2) a sexual partner with an unknown HIV status, (3) had an STI, (4) multiple sexual partners, or (5) sex under the influence of alcohol and/or drugs (8). The recommended regimen is oral Tenofovir disoproxil fumarate/Emtricitabine (TDF/FTC) with a one-month supply provided at initiation followed by three-month supply given at month one (8). Moreover, the guidelines recommend integrating PrEP into existing HIV prevention programs such as sexual and reproductive health (SRH) services (8). In South Africa, PEPFAR has prioritised PrEP for AGYW, due to their disproportionately high HIV incidence (9).

More than 888,000 people are estimated to have initiated PrEP by mid-2023 in South Africa (10). However, the duration of PrEP use in these people is unknown. Given that more than 5 years has elapsed since the accessibility of PrEP in public health facilities in South Africa, we examined PrEP persistence over time among patients attending two health facilities in Gauteng.

## 2 Methods

This a retrospective cohort study of medical record data from government health facilities, one in the Johannesburg District and one in the Sedibeng District, supported by Anova Health Institute as a PEPFAR/USAID implementing partner to the district and provincial Departments of Health. The two sites were selected for convenience, as they had started the PrEP roll-out early and kept PrEP records separately. For patients who initiated PrEP, healthcare workers completed a PrEP Clinical Form, which is kept in patient medical files.

This study includes individuals who initiated PrEP at one of the two health facilities of interest between 2018 and 2022. Data was manually extracted from the PrEP Clinical Form, retrieved from patient folders, and where needed data was also extracted from other documents in patient folders. The following variables were extracted and captured into REDCap (11): date of birth, gender (female, male and transgender), original PrEP initiation date, next visit appointment date, actual visit date, staying on PrEP (yes or no), HIV testing (positive or negative), pregnancy result at PrEP initiation (positive, negative or N/A), and STI screen results at PrEP initiation (positive or negative). We defined PrEP persistence as PrEP use over time from the date of PrEP initiation without an interruption of more than 30 days between prescription refills (12). We used the next visit appointment date to estimate when the supplied prescription would be depleted. Individuals were considered to be no longer persisting on PrEP if there was a gap of more than 30 days between refills or if they completely discontinued PrEP. Time since PrEP initiation is calculated in months, an individual who has completed a 1-month supply will be on PrEP for 1 month since PrEP initiation. Visit number is a count of PrEP clinic visits, for instance, a patient initiating PrEP will be on visit number 1. Although South African guidelines state that 1 month of PrEP should be provided at initiation, we found that in some cases 2 months were provided. This was done to align with clients’ contraceptive appointments. Whether 1 or 2 months of PrEP is provided has implications for measures of persistence, so to account for this variability we included a variable for whether clients received a one-month supply at initiation. We considered PrEP guidelines at initiation to have been followed if the client received a one-month supply of PrEP at their initiation visit.

Analyses were conducted using Stata 14 (StataCorp, College Park, Texas, USA). We used Kaplan–Meier survival curves to illustrate time to overall PrEP discontinuation, and by gender, age, and facility. We excluded individuals from the survival analysis if they had important missing data: the original PrEP initiation date, actual PrEP visit date and next PrEP visit appointment date. We used Cox proportional hazard models to examine factors (gender, age, facility, pregnancy, and PrEP guidelines at initiation) associated with PrEP persistence and adjusted for age, gender and STI screen at initiation. We estimated the hazard ratio of discontinuing PrEP with the 95% Confidence interval (CI).

## 3 Results

We captured 344 (Table 1) files of patients who had initiated PrEP between 2018 and 2022. At the time of PrEP initiation, 18.3% of patients were 15–19 years, 33.1% were 20–24 years, 37.8% were 25–39 years, and 10.5% were ≥40 years. Females accounted for 68.0% (*n* = 234) of those who initiated PrEP, and 135 (39.2%) of patients were adolescent girls and young women (AGYW) between the ages of 15–24 years. Among females who initiated PrEP, 5.8% (*n* = 13) had a positive pregnancy test result at the time of initiation. With regards to STIs, 4.0% (*n* = 13) had a positive STI symptom screen.

**Table 1:** Characteristics at the initiation visit at two study sites, 2018–2022.

Characteristics	Total		Facility A		Facility B	
	*n*	%	*n*	%	*n*	%
Total	344		237		107	
Age (years)						
<15	1	0,29	1	0,42	0	0,00
15–19	63	18,31	62	26,16	1	0,93
20–24	114	33,14	94	39,66	20	18,69
25–39	130	37,79	67	28,27	63	58,88
≥40	36	10,47	13	5,49	23	21,50
Gender						
Female	234	68,02	171	72,15	63	58,88
Male	110	31,98	66	27,85	44	41,12
AGYW (15–24 yrs.)	135	39,24	119	50,21	16	14,95
Pregnancy (females)						
Yes	13	5,83	9	5,52	4	6,67
No	210	94,17	154	94,48	56	93,33
Weight kg, Min – Max (Median)	40–128 (66)		40–112 (63)		44–128 (72)	
Hepatitis B						
Negative	89	36,48	68	38,64	21	30,88
Positive	3	1,23	2	1,14	1	1,47
No Results	152	62,30	106	60,23	46	67,65
STI symptom screening						
Negative	314	96,02	214	94,69	100	99,01
Positive	13	3,98	12	5,31	1	0,99
Visit Number, Min - Max (Median)	1–10 (3)		1–8 (1)		1–10 (3)	

Overall PrEP persistence was 65.7% (*n* = 226) at month 1, and 45.9% (*n* = 158) at month 2 (Table 2). PrEP persistence by gender decreased from 62.8% (females, *n* = 147) and 71.8% (males, *n* = 79) after 1 month to 23.5% (females, *n* = 55) and 34.6% (males, *n* = 38) after 4 months. By age, PrEP persistence decreased throughout, among the 25–39 years it decreased from 73.9% (*n* = 96) after 1 month to 33.9% (*n* = 44) after 4 months of PrEP initiation. With regards to those who followed guidelines at initiation (1 month supply), PrEP persistence decreased from 58.6% (*n* = 170) at 1 month to 26.6% (*n* = 77) at the 4th month. By visit, persistence was 53.5% at follow-up visit 1, and 31.7% at follow-up visit 2.

**Table 2:** Time to PrEP discontinuation over 4 months at two study sites, 2018–2022.

Factors	Initiated (*n*)	Time since initiation (months)							
		1		2		3		4	
		*n*	%	*n*	%	*n*	%	*n*	%
Overall Persistence	344	226	65,70	158	45,93	130	37,79	93	27,03
Gender									
Female	234	147	62,82	102	43,59	85	36,32	55	23,50
Male	110	79	71,82	56	50,91	45	40,91	38	34,55
Age (years)									
15–19	63	36	57,14	23	36,51	21	33,33	12	19,05
20–24	114	67	58,77	36	31,58	31	27,19	22	19,30
25–39	130	96	73,85	78	60,00	60	46,15	44	33,35
≥40	36	27	75,00	21	58,33	18	50,00	15	41,67
One month given at initiation									
Followed	290	170	58,62	126	43,45	99	34,14	77	26,55
Did not follow	54	54	100,00	30	55,56	29	53,70	14	25,93

**Figure 1:**
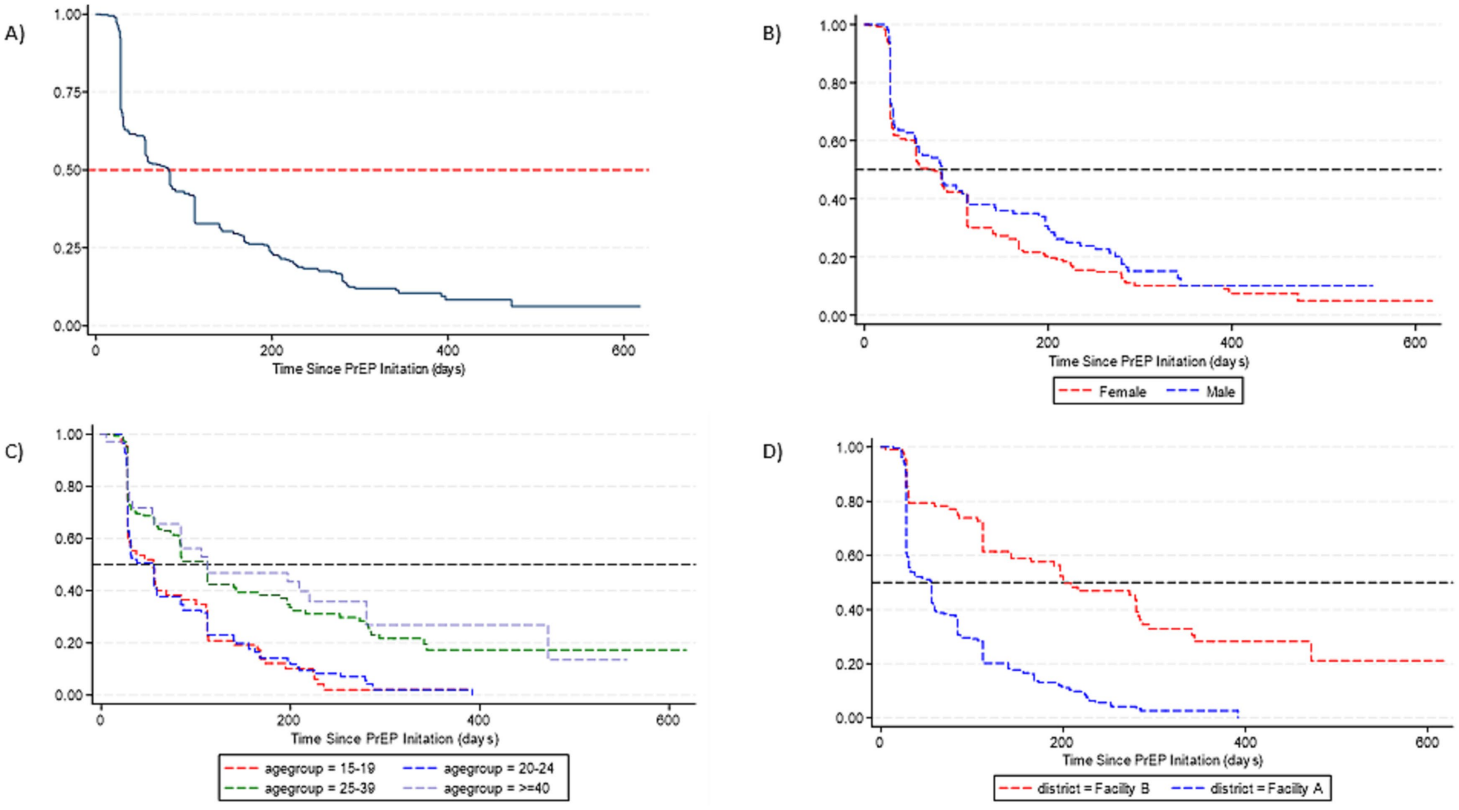
Survival curves for PrEP persistence for individuals who initiated PrEP. **(A)** Time to overall PrEP discontinuation. **(B)** Time to PrEP discontinuation by gender. **(C)** Time to PrEP discontinuation by age. **(D)** Time to PrEP discontinuation by facility.

PrEP persistence decreased over time, particularly after the first month since PrEP initiation (Figure 1) with a median persistence time of 76 days. By gender, median PrEP persistence time was 59 days among females and of 83 days among males. There was no significant difference in overall PrEP persistence by gender (aHR = 1.14, 95%CI: 0.87–1.51) (Table 3). By age groups, the median persistence time was 55 days among 15–19-year-olds, 56 days in the 20–24 years age group, 112 days for the 25–39 years age group, and it was 112 days in those ≥40 years. Individuals who were 25–39 years were less likely to discontinue PrEP (aHR = 0.55, 95%CI: 0.39–0.79) compared to 15–19-years. Additionally, individuals who were ≥40 years were less likely to discontinue PrEP (aHR = 0.52, 95%CI: 0.32–0.86). The median persistence time differed between Facility B (201 days) and Facility A (55 days), with Facility B having a longer survival time (Figure 1). Overall, PrEP discontinuation was more likely in Facility A than in Facility B (aHR = 2.96, 95%CI: 2.10–4.17).

**Table 3:** Factors associated with PrEP persistence at two study sites, 2018–2022.

Factors	HR	95% CI	aHR	95% CI
Gender				
Female	1.20	0.93–1.54	1.14	0.87–1.51
Male	1.00	–	1.00	–
Age (years)				
15–19	1.00	–	1.00	–
20–24	1.00	0.73–1.39	1.05	0.76–1.47
25–39	0.52	0.37–0.73	0.55	0.39–0.79
≥40	0.45	0.28–0.73	0.52	0.32–0.86
Facility				
Facility A	3.22	2.38–4.36	2.96	2.10–4.17
Facility B	1.00	–	1.00	–
Pregnancy at PrEP initiation				
Yes	0.66	0.31–1.42	0.70	0.33–1.50
No	1.00	–	1.00	–
One month given at initiation				
Followed	1.11	0.81–1.52	1.57	1.10–2.24
Did not follow	1.00	–	1.00	–

Adjusted for age, gender and STI at initiation.

In Facility A, PrEP persistence was 58.2% (*n* = 138), and 16.9% (*n* = 40) at month 1 and at month 4 after PrEP initiation (Table 4). While in Facility B, PrEP persistence at month 1 was 82.2% (*n* = 88) and at month 4 was 49.5% (*n* = 53). Among females PrEP persistence at 4 months after initiation was at 17.5% (*n* = 30) and 39.7% (*n* = 25) in Facility A and Facility B, respectively. PrEP persistence decreased throughout the different age groups with persistence being better in the older age groups. PrEP persistence at 4 months after initiation was 15.4% (*n* = 29) in Facility A and 47.1% (*n* = 48) in Facility B among those who received one-month of PrEP at initiation as recommended by the guidelines.

**Table 4:** Time to non-PrEP persistence over 3 months.

Factors	Facility A									Facility B								
	Time since initiation (months)									Time since initiation (months)								
	Initiated (*n*)	1		2		3		4		Initiated (*n*)	1		2		3		4	
		*n*	%	*n*	%	*n*	%	*n*	%		*n*	%	*n*	%	*n*	%	*n*	%
Overall persistence	237	138	58,23	87	36,71	63	26,58	40	16,88	107	88	82,24	71	66,36	67	62,62	53	49,53
Gender																		
Female	171	99	57,89	64	37,43	49	28,65	30	17,54	63	48	76,19	38	60,32	36	57,14	25	39,68
Male	66	39	59,09	23	34,85	14	21,21	10	15,15	44	40	60,61	33	50,00	31	46,97	28	42,42
Age (years)																		
15–19	62	35	56,45	22	35,48	20	32,26	11	17,74	1	1	100,00	1	100,00	1	100,00	1	100,00
20–24	94	54	57,45	28	29,79	23	24,47	15	15,96	20	13	65,00	8	40,00	8	40,00	7	35,00
25–39	67	41	61,19	32	47,76	17	25,37	11	16,42	63	55	87,30	46	73,02	43	68,25	33	52,38
≥40	13	8	61,54	5	38,46	3	23,08	3	23,08	23	19	82,61	16	69,57	15	65,22	12	52,17
Guidelines at initiation																		
Followed	188	89	47,34	61	32,45	37	19,68	29	15,43	102	81	79,41	65	63,73	62	60,78	48	47,06
Did not follow	49	49	100,00	26	53,06	26	53,06	11	22,45	5	5	100,00	4	80,00	3	60,00	3	60,00

PrEP persistence was similar between males and females in Facility A (aHR = 0.97, 95%CI: 0.70–1.34) and Facility B (aHR = 1.17, 95%CI: 0.69–1.99) (Table 5). There was no significant difference in PrEP persistence by age groups in both facilities. Additionally, there were no observed differences in PrEP persistence by pregnancy status. In Facility A, those who received PrEP at initiation as recommended in the guidelines were more likely to discontinue PrEP (aHR = 1.80, 95%CI: 1.23–2.63).

**Table 5:** Factors associated with PrEP persistence in facility A and B, 2018–2022.

Factors	Facility A				Facility B			
	HR	95% CI	aHR	95% CI	HR	95% CI	aHR	95% CI
Gender								
Female	0.91	0.68–1.23	0.97	0.70–1.34	1.19	0.72–1.98	1.17	0.69–1.99
Male	1.00	–	1.00	–	1.00	–	1.00	–
Age (years)								
15–19	1.00	–	1.00	–	1.00	–	1.00	–
20–24	1.11	0.79–1.55	1.13	0.80–1.60	0.87	0.11–6.74	0.92	0.12–7.19
25–39	0.92	0.64–1.233	0.94	0.64–1.38	0.46	0.06–3.38	0.45	0.06–3.40
≥40	0.81	0.42–1.55	0.81	0.41–1.60	0.48	0.07–3.71	0.54	0.07–4.23
Pregnancy at PrEP initiation								
Yes	0.54	0.22–1.31	0.55	0.22–1.35	1.10	0.26–4.64	1.24	0.29–5.33
No	1.00	–	1.00	–	1.00	–	1.00	–
Guidelines at initiation								
Followed	1.65	1.17–2.31	1.80	1.23–2.63	1.13	0.35–3.60	1.23	0.37–4.14
Did not follow	1.00	–	1.00	–	1.00	–	1.00	–

Adjusted for age, gender and STI at initiation.

## 4 Discussion

This analysis provides evidence of how long patients chose to stay on PrEP at two sites that have integrated PrEP provision into primary health care services in South Africa. We found that overall 37.8% remained on PrEP at month 3 and 27.0% at month 4 after initiation. In a study of heterosexual men in South Africa (using the same national guidelines) 40% returned for the second follow-up visit at month four. Of note, almost a third of men returned more than 30 days later than scheduled, and there were no associations between demographic characteristics and persistence (13). Our estimate was also lower than PrEP persistence (87% at month 3) in a study among women conducted in Uganda (14). Unlike our study, the Ugandan study was conducted only among women with partners living with HIV, while our study included participants who initiated PrEP, regardless of their partner’s HIV status. Additionally, PrEP persistence was high (80% at month 3) in a PrEP trial among women in Durban (15). However, PrEP persistence declined drastically post-trial (47.1%) because of PrEP accessibility challenges (16). Although PrEP is freely available at South African health facilities, there are accessibility challenges including transport and opportunity costs and fear of judgement requesting SRH services (16).

In our analysis, more females had initiated PrEP, with 39.2% of total initiations being in AGYW. AGYW are a target population for PrEP, with targets assigned by PEPFAR, and much of the facility-based demand creation work targets young women, for example through peer supporters. It is clear, however, that there is demand for PrEP among females and males as males made up 32.0% of our enrollments. Participants older than 24 years accounted for 48.3% of those who initiated PrEP. Similar to our findings, a study conducted in the Eastern Cape found low PrEP uptake among adolescents and young adults (16–24 years) (17). Moreover, we found that age older than 24 years (25–39 year and ≥40 years) was associated with longer persistence compared to the 15–19-years age group. Prior research in the United States supports that there is poorer persistence in younger age groups (18–24 years) than in older age groups (12). PrEP services for AGYW in the community have been shown to be feasible and acceptable, and may be a preferred alternative to facility based services (18).

At the time of data collection, systems to provide PrEP to pregnant women had not been integrated into antenatal services. However, 5.8% of our female enrollments were in pregnant women, suggesting that there would be a demand for PrEP among pregnant women if it were to be offered consistently. Previous studies have found that providing PrEP to pregnant and breastfeeding women could decrease vertical transmission (19), and higher continuation can be achieved in pregnant compared to other women (20). At the time of writing, PrEP was being provided in antenatal services, although accessibility barriers remain and should be addressed to realize the potential of this key prevention measure (21).

Our analysis also showed those who received PrEP at initiation as recommended in the guidelines were more likely to discontinue PrEP compared to those for whom the guideline recommendation was not followed. This is because providing 2 months of PrEP at initiation means a client cannot be considered to have discontinued until after their month two return visit, artificially inflating their persistence. By month 4, persistence was similar regardless of months dispensed at initiation.

Positive STI screening rates were very low considering the high prevalence and incidence of STIs in South Africa (22, 23). This highlights the inadequacy of symptom screening to identify STIs in high risk settings, and supports the recommendation that PrEP users be offered diagnostic STI screening (i.e., diagnostic tests in the absence of symptoms) at initiation and annually thereafter (24).

Based on the captured files, Facility A initiated higher numbers of people onto PrEP (*n* = 237) than Facility B (n = 107), and PrEP persistence differed substantially by site, with Facility B having longer persistence compared to Facility A. Since age and gender have been adjusted for in the model, we believe the differences are related to service delivery rather than service users. The sites followed the same guidelines and procurement policies, but there were likely service delivery differences in counselling or recruitment of clients for PrEP that could have led to differing persistence. For example, Facility A did not have a dedicated Adolescent and Youth Friendly Services nurse for much of the study period, meaning that clients attending for PrEP follow-up visits were seen in mainstream queues, leading to longer waiting times, and care that was less tailored to young people specifically. Adolescent-friendly services have been shown to improve levels of comfort and engagement with PrEP services (18). Facility B, however, had a dedicated counsellor who reminded PrEP users of their upcoming appointment, supporting persistence. We believe these service delivery factors played an important role in PrEP persistence. The most effective way to deliver PrEP services embedded in primary health care facilities is an important avenue for further research.

As a limitation, PrEP persistence in our study was measured by number of months of PrEP supply given at each visit and was not measured by pill count or detecting levels of PrEP in blood patient’s blood. Moreover, the frequency of PrEP supply varied instead of following the government recommendation of 1-month supply followed by 3-monthly supplies (8). The inconsistency stemmed from guidelines recommending that PrEP is integrated into existing SRH services (8). Thus, most patients accessing SRH service such as family planning would have their PrEP visits aligned with their family planning visits. Moreover, our study included a small number of sites. Lastly, given that data was retrospectively extracted from medical records there were some records with incomplete or incorrectly captured forms. Our data capturing was limited to forms that were legible, complete and/or correctly captured. A strength of our study was the use of longitudinal data of patients receiving PrEP at public health facilities.

## 5 Conclusion

Our knowledge of PrEP usage patterns and persistence remains limited despite the amount of time that has elapsed since PrEP was made accessible in public health facilities in South Africa. We have shown that most people stop taking PrEP before their second follow-up visit. Also, individuals >24 years had better PrEP persistence than individuals 15–19 years. PrEP uptake is heavily driven by HIV risk perception and awareness (25). People are motivated to continue taking PrEP to protect themselves from acquiring HIV and thus might stop taking PrEP when they no longer feel at risk (26) and/or perhaps due to pill burden and fear of disclosing (27). Service delivery factors also appear to have a substantial effect on PrEP persistence. Health facilities should continue to make PrEP accessible by integrating PrEP with existing services, and considering differentiated PrEP services for different populations.

## Data Availability

The raw data supporting the conclusions of this article will be made available by the authors, without undue reservation.
